# Rare Mononuclear Lithium–Carbene Complex for Atomic Layer Deposition of Lithium Containing Thin Films

**DOI:** 10.1002/anie.202513066

**Published:** 2025-09-04

**Authors:** Jorit Obenlüneschloß, Nils Boysen, Karl Rönnby, Arbresha Muriqi, Volker Hoffmann, Carlos Abad, Detlef Rogalla, Ulrike Brokmann, Edda Rädlein, Michael Nolan, Anjana Devi

**Affiliations:** ^1^ Inorganic Materials Chemistry Ruhr University Bochum Universitätsstr. 150 44801 Bochum Germany; ^2^ Fraunhofer IMS Finkenstr. 61 47057 Duisburg Germany; ^3^ Tyndall National Institute, Lee Maltings University College Cork Cork T12 R5CP Ireland; ^4^ Leibniz Institute for Solid State and Materials Research (IFW) Dresden e.V. Helmholtzstr. 20 01069 Dresden Germany; ^5^ Bundesanstalt für Materialforschung und ‐prüfung (BAM) 12205 Berlin Germany; ^6^ RUBION Ruhr University Bochum Universitätsstr. 150 44801 Bochum Germany; ^7^ Group of Inorganic–Nonmetallic Materials Technische Universität Ilmenau Gustav‐Kirchhoff‐Str. 6 98683 Ilmenau Germany; ^8^ Chair of Materials Chemistry, TU Dresden Bergstr. 66 01069 Dresden Germany

**Keywords:** Atomic layer deposition, Carbene ligands, Lithium, Precursor, Silicate

## Abstract

Lithium is the core material of modern battery technologies and fabricating the lithium‐containing materials with atomic layer deposition (ALD) confers significant benefits in control of film composition and thickness. In this work, a new mononuclear N‐heterocyclic carbene (NHC) stabilized lithium complex, [Li(^tBu^NHC)(hmds)], is introduced as a promising precursor for ALD of lithium‐containing thin films. Structural characterization is performed, comparing density functional theory (DFT) and single‐crystal X‐ray diffraction (SC‐XRD), confirming a rare mononuclear structure. Favorable thermal properties for ALD applications are evidenced by thermogravimetric analysis (TGA). The compound exhibits a low melting point, clean evaporation, and its volatility parameters are encouraging compared to other lithium precursors. ALD trials using [Li(^tBu^NHC)(hmds)] with ozone demonstrate its effectiveness in depositing LiSi*_x_* O*_y_* films. The ALD process exhibits a saturated growth per cycle (GPC) of 0.95 Å. Compositional analysis using Rutherford backscattering spectrometry/nuclear reaction analysis (RBS/NRA), X‐ray photoelectron spectrometry (XPS), and glow discharge optical emission spectrometry (GD‐OES), confirms the presence of lithium and silicon in the expected ratios. This work not only presents a new ALD precursor but also contributes to the understanding of lithium chemistry, offering insights into the intriguing coordination chemistry and thermal behavior of lithium complexes stabilized by NHC ligands.

## Graphical abstract

A new N‐heterocyclic carbene (NHC) stabilized lithium complex is introduced as a thermally stable atomic layer deposition (ALD) precursor. It features a rare mononuclear structure, suitable volatility, and a low melting point of 55 °C. ALD trials yielded lithium silicate films. This study opens new directions for NHC‐ligand chemistry in thin‐film deposition applications.

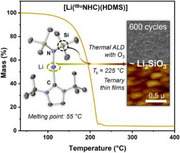

## Introduction

Lithium, from the Greek word “lithos”, the stone as it was discovered as an unexpected impurity in mineral rocks, is among the most coveted materials over recent years.^[^ ^1^ ^]^ This arises from the vast adaptation of lithium‐ion batteries (LIBs) for energy storage in small hand‐held devices, electric vehicles, and grid‐scale storage, with demand for lithium expected to grow substantially.^[^ ^2^, ^3^, ^4^ ^]^

Atomic layer deposition (ALD) has proven to be a leading technique for fabricating thin films with Angstrom‐level precision and ALD can be used to produce various LIB components as previously described.^[^ ^5^, ^6^, ^7^ ^]^ ALD allows to design, fabricate, and implement LIB components such as the cathodes, anodes, and solid electrolytes in new ways or to precisely tune interfacial properties.^[^ ^8^ ^]^ Lithium‐containing materials have been realized by ALD for cathode materials (e.g., lithium‐nickel‐silicon‐oxide; using [Li(hmds)]_3_, [NiCp_2_], O_2_ plasma),^[^ ^9^ ^]^ anodes (e.g., Li_4_Ti_5_O_12_; with LiOtBu, TTIP, H_2_O),^[^ ^10^ ^]^ as the electrolyte (e.g., lithium silicates; with LiOtBu, tetraethylorthosilane, H_2_O),^[^ ^11^ ^]^ as well as surface modifications (e.g., LiAlO_2_; with LiOtBu, TMA, H_2_O).^[^ ^12^ ^]^ Especially, for the rapid charging of such batteries, 3D micro‐structuring and an increase of internal surface area are beneficial.^[^ ^13^ ^]^ Hence, all solid‐state LIBs (ASSLIBs) can benefit significantly from the efficient implementation of ALD for their components.^[^ ^14^ ^]^

However, lithium, as part of the alkali metals alongside sodium, potassium, rubidium, and cesium, is difficult to work with in ALD. These group 1 elements have a set of properties that make them hard to access through precursor chemistry, and the resulting materials have challenging properties. Even when not in their highly reactive elemental state, alkali metals are still very oxophilic and tend to form hydroxides or carbonates on the surface, altering the desired material properties.^[^ ^15^, ^16^ ^]^ For all alkali metal precursors (excluding Rb and Cs due to the scarcity of available precursor‐related studies), clustering of the molecules into multinuclear complexes is an issue as it leads to relatively high sublimation temperatures. This is caused by the polar metal‐ligand bonds these electropositive elements exhibit.^[^ ^17^, ^18^ ^]^ Further, the growth behavior is expected to be governed by Coulombic interactions rather than covalent bonding as often assumed for ALD reactions.

To counter the clustering effect, sterically demanding ligands have been used for these elements and especially lithium, e.g. *tert*‐butoxide (OtBu), 2,2,6,6‐tetramethyl‐3,5‐heptanedionate (thd), hexamethyldisilazide (hmds), and most recently trimethylsilanolate (TMSO).^[^ ^19^ ^]^ For lithium, these and other precursors have been extensively tested since 2009, when a broad range of precursor candidates were investigated. These include the β‐diketonate [Li(thd)] precursor, which is mostly used compared to precursors with alkoxide, cyclopentadienyl, n‐butyllithium, and the dicyclohexylamide ligands.^[^ ^20^ ^]^ Many efforts have been made to establish new lithium precursor chemistry, which include, for instance, the less volatile [Li(hfac)], the equally low volatile trifluoroacetate, and the more suitable trimethylsilanolate, which still required a source temperature of 165 °C.^[^ ^21^, ^22^ ^]^

Nonetheless, an often favored precursor is the [Li(hmds)]_3_ because of its lower melting point of 70 °C and favorable volatility (source temperature as low as 60 °C).^[^ ^22^, ^23^ ^]^ Also, the range of accessible materials from this precursor makes it one of the most enticing choices. In addition to its dual‐source behavior that has been exploited for lithium silicate and oxide films,^[^ ^23^, ^24^ ^]^ it has been utilized for the ALD of lithium nitride, carbonates,^[^ ^25^ ^]^ and the solid‐state electrolyte LIPON.^[^ ^26^ ^]^ Nonetheless, its dinuclear aggregation in the gas phase could lead to mixed order kinetics, where a mononuclear complex would be highly advantageous as it is expected to be more reactive.^[^ ^27^, ^28^ ^]^

In our study, we explored a new way to modify this promising core unit, Li(hmds), to improve upon its already favorable properties. As showcased for other elements in oxidation state + I, which tend to form dinuclear complexes as seen for copper or silver in the case of the acetamidinate precursors,^[^ ^29^, ^30^ ^]^ charge‐neutral σ‐electron donating ligands can be utilized to fill the coordinative undersaturation and sterically shield the metal center to create a mononuclear compound. In addition to the σ‐electron donation, π‐electron backdonation from the metal to the ligand can impart additional stability for these d‐block metals.^[^ ^31^ ^]^ Noteworthy, work in this direction was performed by Coyle et al. and Boysen et al. on copper and silver, combining the respective hexamethyldisilazide and diketonate compounds with the neutral electron‐donating N‐heterocyclic carbene (NHC) ligand, resulting in promising ALD precursors.^[^ ^32^, ^33^, ^34^ ^]^

Adapting a similar approach to lithium is a challenge as lithium only possesses filled s orbitals and lacks the d orbitals involved in the π‐electron backdonation, which is a feature typical of carbene‐metal interactions. However, the strongly nucleophilic character of the NHC should allow the formation of stable bonds even with the lack of π‐electron backdonation.^[^ ^35^ ^]^ This is proven for lithium with NHC‐stabilized Li‐cyclopentadienes by Arduengo et al., who have pioneered the synthesis and isolation of NHCs as early as the 1990s.^[^ ^36^, ^37^, ^38^ ^]^ This also remains the only true example of a mononuclear lithium mono‐carbene complex to this day. While homoleptic but di‐nuclear carbene‐lithium complexes have since been realized,^[^ ^35^, ^39^ ^]^ seemingly monomeric compounds could only be realized with bulky bis‐NHC ligands and the addition of coordinating solvents or with positively charged Li‐NHC complexes with weakly aggregated anions.^[^ ^40^, ^41^ ^]^ One monomeric example of a mononuclear lithium silyl amide NHC complex can be found in the work of Koch et al., who utilized a bidentate bis‐NHC with bulky diisopropylphenyl side chains.^[^ ^42^ ^]^ Interestingly, when a sterically demanding alkoxide (lithium (2,4,6‐trimethyl)phenolate) was used, a bridged dinuclear compound was formed, which might be expected if the lithium *tert*‐butoxide was chosen as the core unit instead.^[^ ^43^ ^]^ The success of the donor ligand strategy in general has been shown by the use of coordinating and even chelating ligands for monomeric lithium complexes, too, e.g., the Li‐cyclopentadienes combined with the η^2^‐TMEDA ligand.^[^ ^44^ ^]^

Lithium silylamides are widely used reagents.^[^ ^45^ ^]^ They find use as Brønsted bases and are often utilized to form amide complexes of other metals via salt metathesis reactions.^[^ ^46^ ^]^ The kinetics of these reactions are dependent on the aggregation state, which is often complicated by the equilibrium between different aggregation states.^[^ ^47^, ^48^ ^]^ Thus, to obtain lithium silylamides in a mononuclear state has been of high interest. Despite multiple examples of stable NHC‐coordinated lithium compounds, their use as precursors for chemical vapor deposition (CVD) and ALD has not been tested so far. Especially, because of their highly reactive nature, finding volatile and yet thermally stable complexes remains a significant challenge. To tackle this challenge, we further explored the NHC chemistry, and herein, we present the successful synthesis of a mononuclear NHC‐stabilized lithium silylamide complex, namely (1,3‐di‐*tert*‐butyl‐imidazolin‐2‐ylidene) lithium hexamethyldisilazide [Li(^tBu^NHC)(hmds)], and evaluation of its properties. Structural analysis is performed by means of density functional theory (DFT) and single‐crystal X‐ray diffraction (SC‐XRD), followed by thorough thermogravimetric analysis (TGA) to assess its volatility and thermal stability. Based on the promising physico‐chemical properties, [Li(^tBu^NHC)(hmds)] was effectively implemented as an ALD precursor in combination with ozone (O_3_) as co‐reactant for the growth of lithium silicate, LiSi*_x_* O*_y_*, films, which can find use as a solid electrolyte.^[^ ^11^ ^]^ This study explores the family of NHC‐stabilized Li complexes that can meet the desired properties needed for vapor phase deposition processes.

## Results and Discussion

### Precursor Synthesis and Characterization

**Scheme 1:**
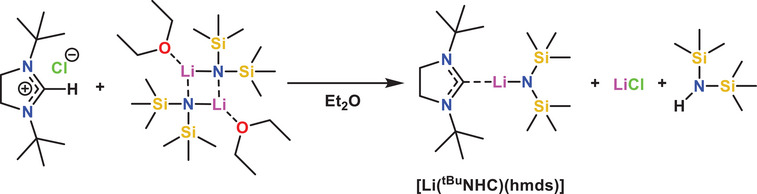
Synthesis route adapted for [Li(^tBu^NHC)(hmds)].

The synthesis of [Li(*^t^* ^Bu^NHC)(hmds)] adapts a similar “one‐pot” approach as reported for the structurally analogous copper and silver complexes.^[^ ^33^, ^49^ ^]^ In the reaction vessel, solid Li(hmds) etherate dimer and 1,3‐di‐*tert*‐butylimidazolium chloride salt are combined in diethyl ether to yield the target compound (Scheme 1) upon crystallization or sublimation under reduced pressure (60 °C, 10^−2^ mbar). One Li(hmds) unit serves as a strong base to deprotonate the imidazolium salt and generate the free carbene, which coordinates to the remaining Li(hmds). The resulting colorless compound was found to be highly air‐ and moisture‐sensitive. It should be noted that synthesis of [Li(*^t^* ^Bu^NHC)(hmds)] can have a disadvantage in terms of atom economy when compared to the parent compound [Li(hmds)]_3_, which is also used as an ALD precursor. Nonetheless, the introduction of the NHC ligand enabled the lowering of the melting point, as discussed below, and generally opens interesting possibilities for the exploration of other lithium coordination complexes based on NHCs. Analysis of [Li(*^t^* ^Bu^NHC)(hmds)] by ^1^H and ^13^C NMR (Figure SI 1) revealed a high degree of similarity to the analogous Cu and Ag compounds.^[^ ^33^, ^49^, ^50^ ^]^ Equal stoichiometry of the ligands is confirmed by three distinct signals with matching integral ratios, further confirming a symmetric structure for the compound with both the *tert*‐butyl and the trimethyl silyl groups being magnetically equivalent. Most noteworthy is the carbenic carbon atom with its signal shifted downfield at 213.7 ppm, when compared with its silver and copper analogues (201.6 and 205.1 ppm).^[^ ^49^ ^]^ As an increased extent of π‐electron backdonation has previously explained an upfield shift in the silver and copper complexes, it is foreseeable to observe the opposite effect for lithium, which lacks any π‐electrons. Without such π‐electrons, the partial positive charge at the carbenic carbon cannot be compensated. The signal of the fully deshielded carbenic carbon atom in the free carbene has been reported at 238 ppm.^[^ ^51^ ^]^ For similar alkali metal complexes using different NHCs, the observed shifts are in a comparable region.^[^ ^52^, ^53^ ^]^

### Investigation of Thermal Properties

**Figure 1:**
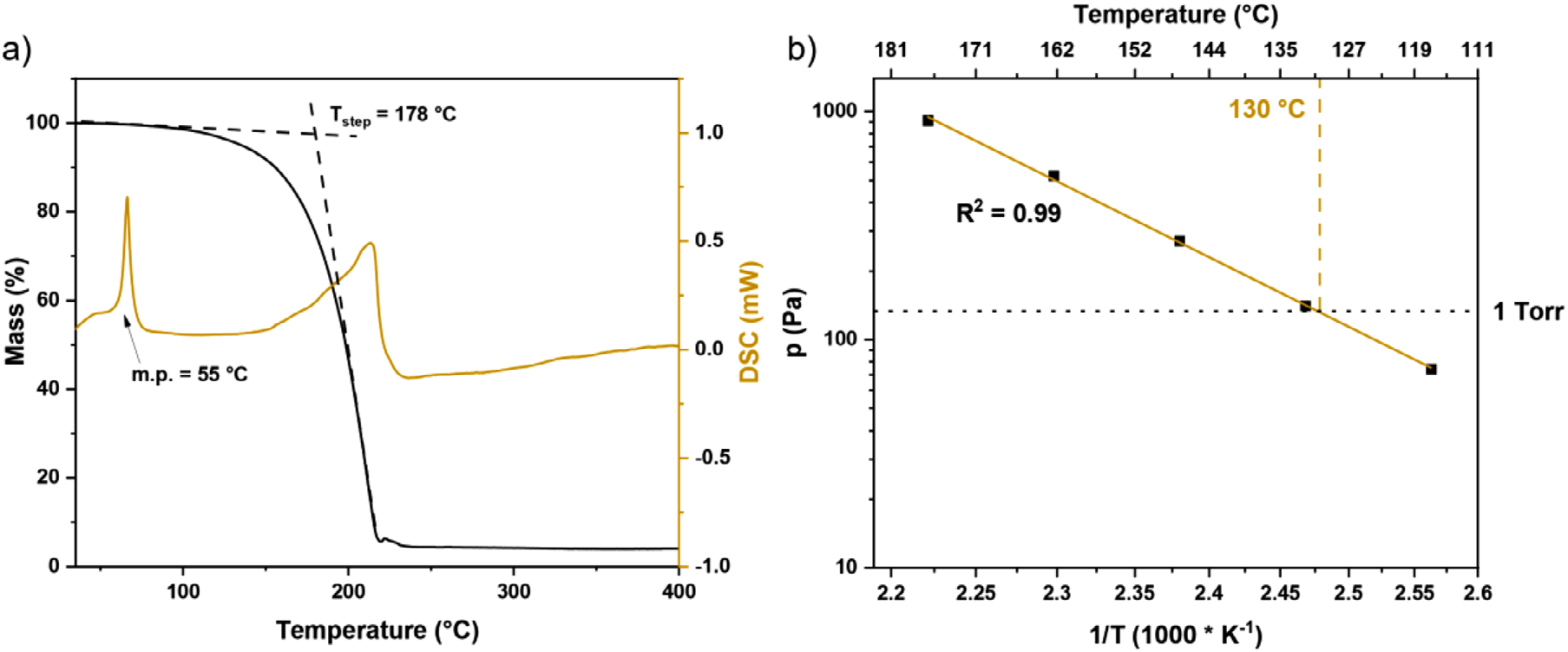
a) TGA of [Li(^tBu^NHC)(hmds)] and corresponding DSC curve. Dashed lines give guidance for the step temperature; additionally, the melting point is highlighted in the DSC curve. b) Vapor pressure Clausius–Clapeyron plot of [Li(^tBu^NHC)(hmds)]. Dashed lines are to guide the eye for the 1 Torr vapor pressure temperature.

The thermal properties of [Li(^tBu^NHC)(hmds)] were investigated in detail to assess its suitability as a precursor for ALD. Thus, TGA was performed, and subsequently, the vapor pressure was determined. Figure 1a shows that the compound exhibits a clean one‐step evaporation with a low residual mass (∼4%), which indicates a high thermal stability in the temperature range of its evaporation. This is especially remarkable as such low residual masses are unusual for highly reactive compounds. Good volatilization is also evidenced by the onset (1% mass loss temperature) of 88 °C and the step temperature determined with the tangent method as 178 °C. This compares very well to other lithium precursors reported in the literature (Table 1). Especially, the melting point at 55 °C estimated by differential scanning calorimetry (DSC) is lower than that of the parent compound [Li(hmds)]_3_ (70 °C). While evaporation temperatures in an ALD reactor for both compounds are usually higher and above the melting points of either, [Li(^tBu^NHC)(hmds)] confirms the trend of asymmetric heteroleptic compounds exhibiting lower melting points than their homoleptic counterparts. In terms of volatility, both compounds have a very similar onset of evaporation temperature, despite [Li(^tBu^NHC)(hmds)] containing one additional large ligand. This can be reasoned with the agglomeration behavior of the [Li(hmds)]_3_ forming trinuclear complexes in the solid state and dinuclear complexes as volatile species in the gas phase, which are close in molecular weight to [Li(*^t^* ^Bu^NHC)(hmds)] ([Li(hmds)]_2_ 334.7 g mol^−1^, [Li(^tBu^NHC)(hmds)] 349.6 g mol^−1^).^[^ ^54^, ^55^ ^]^ Readers are referred to the work of Hämäläinen et al. for the TGA of Li(hmds), even though direct comparison is difficult due to different instrumentation and settings used.^[^ ^22^ ^]^

**Table 1:** Thermal parameters of commonly used lithium ALD precursor compounds.

Precursor	Step T (°C)	Melting point (°C)	Source
[Li(^tBu^NHC)(hmds)]	178	55	This work
Li(thd)	275	267	[20]
Li(hmds)	175	71 [24]	[22]
LiOtBu	250	150	[22]
Li(TMSO)	190	> 140	[21]

The vapor pressure of [Li(^tBu^NHC)(hmds)] was assessed, performing a stepped isothermal TG measurement. In Figure 1b, the resulting Clausius–Clapeyron plot is displayed correlating the vapor pressure to the temperature of the sample. 1 Torr of vapor pressure is reached at 130 °C. The combination of low melting point, high thermal stability despite being highly reactive, and suitable vapor pressure renders [Li(^tBu^NHC)(hmds)] a promising ALD precursor.

### Structural Investigation by DFT Studies and SC‐XRD

**Figure 2:**
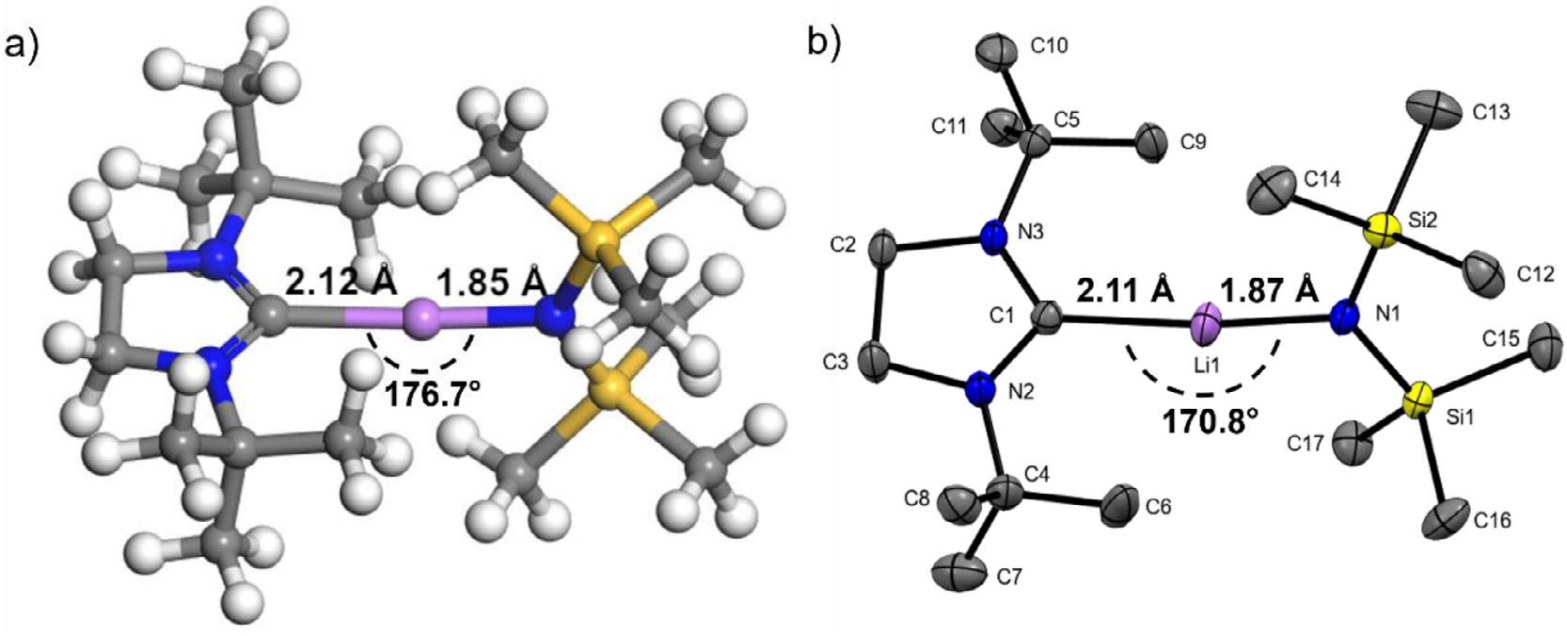
a) DFT relaxed atomic structure and b) molecular crystal structure of [Li(^tBu^NHC)(hmds)], in (b) hydrogen atoms are omitted for clarity, while the thermal ellipsoids are shown with 50% probability. Bond lengths and angles of interest are given in the figure.

DFT structure optimization reveals that the [Li(^tBu^NHC)(hmds)] complex adopts a similar, almost linear structure previously found for the analogous heteroleptic complexes with copper(I) and silver(I). The structure from DFT yields a Li─C distance of 2.12 Å and a Li─N distance of 1.85 Å, which are similar to the experimental structure and suggest different bond strengths between the Li center and the two ligands (Figure 2).

Computing the bond dissociation energies (for cleaving the respective ligand first) clearly shows this difference. The Li─N bond dissociates with an energy cost of 390.1 kJ mol^−1^, and the carbene C─Li bond dissociates with a lower energy cost of 114.1 kJ mol^−1^. This clear difference in bond strengths is expected since the Li─N bond has a strong ionic character, whereas the carbene–lithium bond is rooted in dative covalent interactions. This reactivity estimate gives a first hint at possible deposition pathways. Cleavage of the NHC is more likely to happen first, which would allow for some nucleophilic surface sites to coordinate to the remaining Li(hmds) on a substrate. The dissociation energy for cleavage of the hmds ligand for [Li(^tBu^NHC)(hmds)] is similar to the computed values for the structurally analogous Cu(I) and Ag(I) (446 kJ mol^−1^ and 341 kJ mol^−1^) complexes.^[^ ^49^ ^]^

The DFT structure and geometry are in accordance with the SC‐XRD findings described below, also showing the expected monomeric linear structure (Figure 2). [Li(^tBu^NHC)(hmds)] crystallizes in space group *P*2_1_/*n* in the monoclinic system. Further, a much stronger deviation from the very linear NHC Cu and Ag complexes is seen, and the C–Li–N angle is much steeper, with 170.8°. Although the DFT structure is slightly more linear with the C–Li–N angle at 176.7°, it nonetheless displays a deviation from linearity. The inability of π‐backdonation for Li cannot force the NHC ligand into a strict linear geometry to allow for the required orbital overlap. Instead, only the spherical s‐orbital that is recipient of the σ‐electrons of the carbene governs the interaction and doesn't dictate any directionality. The essentially linear configuration is attributable to the sterically demanding substituents, the *tert*‐butyl groups, and the trimethylsilyl groups. If less bulky groups were chosen, the formation of a dimer without such a linear configuration becomes likely.

A further comparison to the Ag and Cu NHC (hmds) complexes reveals a more imbalanced geometry. The experimental structure shows that the carbene C─Li bond is significantly longer (2.11 Å) than the Li─N bond (1.87 Å) in the case of [Li(^tBu^NHC)(hmds)], while the analogous Cu and Ag compounds show more symmetrical metal‐C and metal‐N distances. This is reflected in the spread of the computed bond dissociation energies as well as in the shift of the ^13^C NMR signal of the carbene carbon.

For the trimeric parent compound [Li(hmds)]_3_, a Li─N bond length of approximately 2 Å was observed. Thus, the coordinating NHC ligand stabilizes the Li─N bond. This can prove beneficial for the first surface adsorption step in an ALD process. Whereas for Li(hmds), cleavage of any bond is equally likely, here, a clear preference for the NHC as the first leaving ligand is expected.

Compared to the closest structurally related compound keeping the NHC ligand, the original NHC‐stabilized Li‐cyclopentadiene, the remarkable role of the silylamide is apparent as it does not possess a delocalized π‐electron system, unlike the cyclopentadiene. It also lacks in steric bulk compared to the tris‐trimethylsilyl substituted cyclopentadiene used by Arduengo et al.^[^ ^38^ ^]^

**Figure 3:**
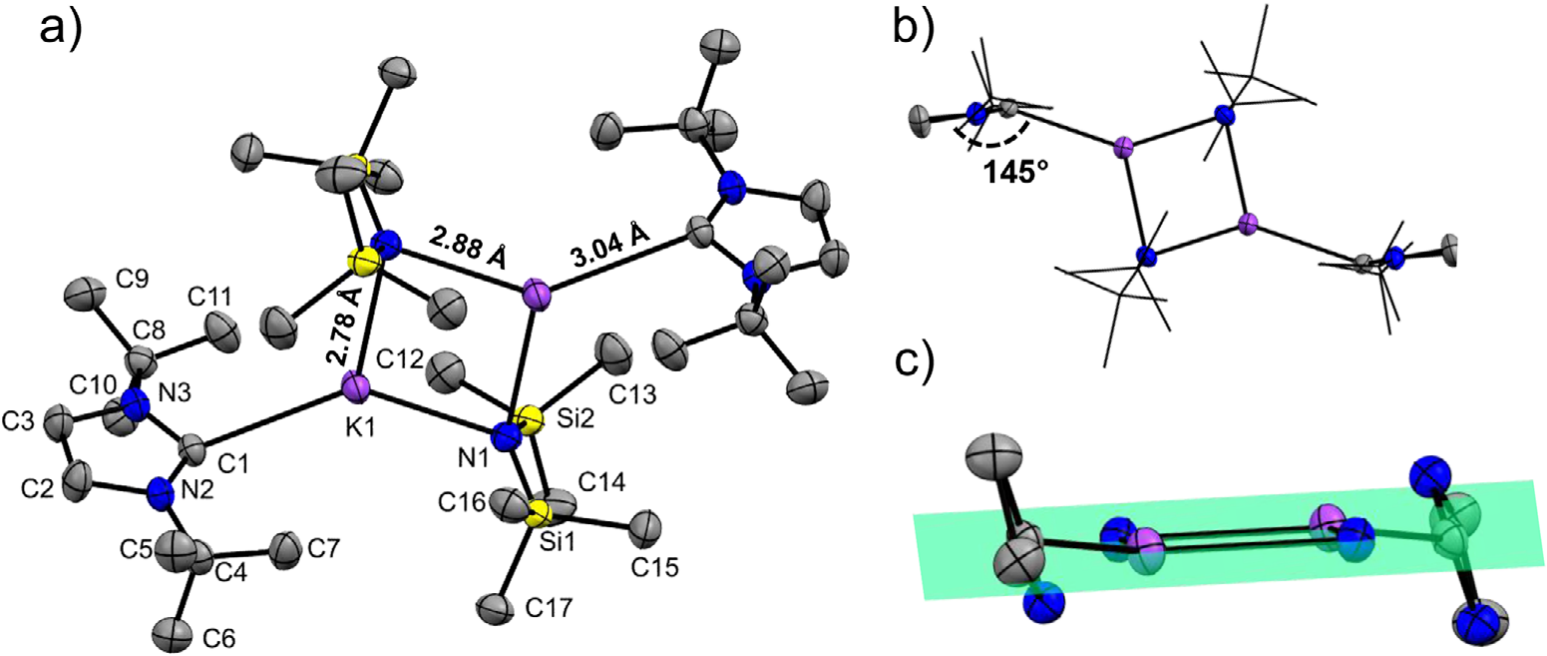
a) Molecular crystal structure of [K(^tBu^NHC)(hmds)]_2_. Hydrogen atoms, as well as symmetry equivalent atom names, are omitted for clarity, while the thermal ellipsoids are shown with 50% probability. The molecular crystal structure is arranged to highlight b) the tilting of the NHC ring, and c) the twisting of the NHC ring with respect to the ring of the four central atoms. Alkyl chains are depicted in wireframe style in (b), and the *tert*‐butyl groups are omitted for clarity in (c). Bond lengths and angles of interest are given in the figure.

When the structure of [Li(^tBu^NHC)(hmds)] is compared to the single crystal structure of its potassium analogue [K(^tBu^NHC)(hmds)]_2_, synthesized following the same procedure for comparison without further in‐depth analysis by other methods, significant differences become apparent (Figure 3a–c). Unlike the mononuclear lithium compound, the potassium variant adopts a dinuclear structure with the potassium atoms bridged by the nitrogen atoms of the silylamide ligands, forming a distorted parallelogram. The NHC ligands coordinate to the potassium on either side. A similar structure was first reported by Alder et al., utilizing an analogous carbene with a six‐membered ring.^[^ ^52^ ^]^ Dinuclear structures for sodium and potassium silylamide bis‐NHC complexes, in contrast to the monomeric lithium counterpart, were also reported by Koch et al.^[^ ^42^ ^]^ It is especially noteworthy to discuss the orientation of the NHC toward the potassium; while [Li(^tBu^NHC)(hmds)] is found to have a near linear coordination and further the Li─NHC bond is in‐plane with the NHC ring, the NHC ring in [K(^tBu^NHC)(hmds)]_2_, however, is tilted and twisted. It adopts a 145° angle with respect to the K─NHC bond, which translates to a 35° pitch angle and is further twisted relative to the plane of the central potassium and nitrogen atoms, the yaw angle. More discrepancies are found in the bond lengths of [K(^tBu^NHC)(hmds)]_2_: the K─N bonds are 2.78 Å or 2.88 Å, and the K─C bond is 3.04 Å in length, significantly longer than for the Li variant. One explanation is found in the ionic radius of potassium, which is close to three times that of lithium.^[^ ^56^ ^]^ Similar bond lengths in the range of 2.84 to 3.16 Å have been observed for related potassium NHC complexes, but the interaction mode of the NHC and the potassium has been speculated to be of ionic dipole character only.^[^ ^52^, ^57^, ^58^ ^]^ Due to its large size, potassium also seems to be susceptible to interactions with the δ carbon to saturate its coordination sphere.^[^ ^58^ ^]^ The twisting of the NHC is likely caused by this phenomenon as the δ carbon to potassium distances are nonequivalent for the two *tert‐*butyl sidechains and near possible interaction at 3.3 Å.

Summarizing, the profound structural differences between the Li and K NHC(hmds) complexes highlight the special case of thermally robust NHC‐alkali metal interaction that is found in the [Li(^tBu^NHC)(hmds)]. It also highlights its monomeric structure as a key point in achieving the observed volatility rivaling that of [Li(hmds)]_3_.

### ALD of Li*_x_* Si*_y_* O*_z_* and Film Characterization

**Figure 4:**
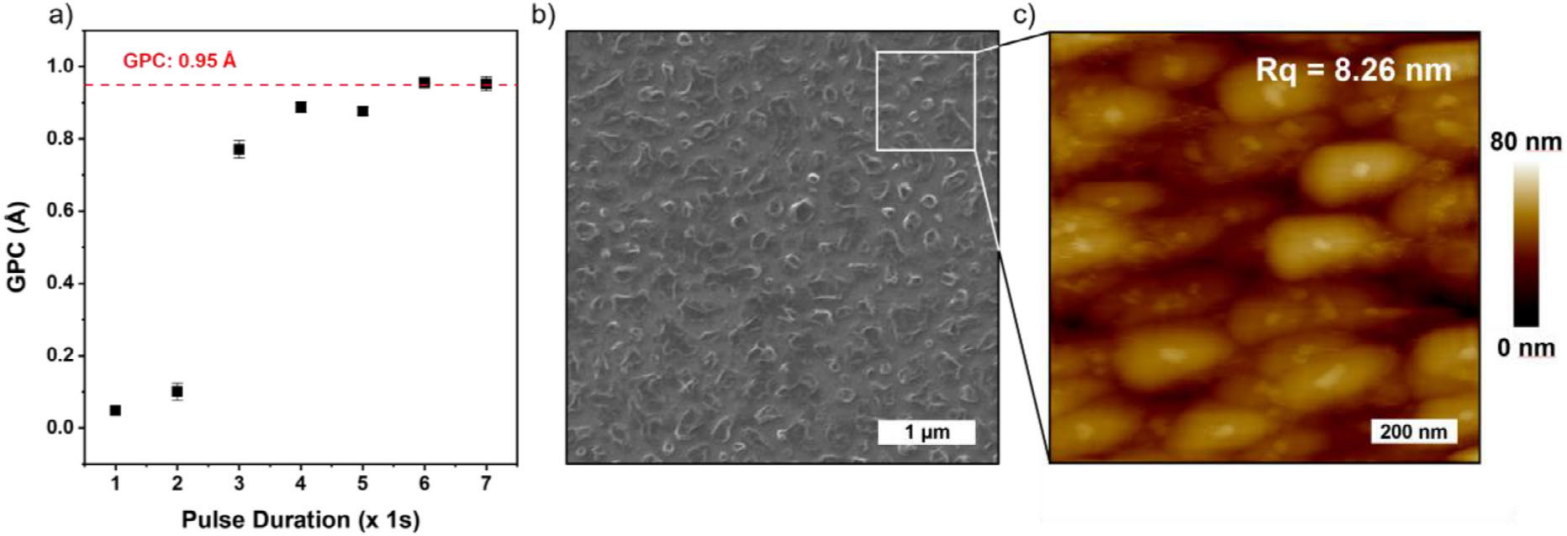
a) Saturation, GPC versus number of 1 s pulses for films grown with 600 cycles. The saturated GPC value of 0.95 Å is highlighted with a dashed line. b) SEM and c) AFM images of films grown by ALD with [Li(^tBu^NHC)(hmds)] and ozone. The zoom illustration serves to visualize the scale only. Approximate film thickness of 50 nm determined by ellipsometry.

Based on the highly promising thermal properties and the preferred monomeric nature, [Li(^tBu^NHC)(hmds)] was tested for ALD. Depositions were performed on Si substrates with ozone as co‐reactant to compare with previous studies that have used [Li(hmds)]_3_ with ozone.^[^ ^24^, ^27^ ^]^ A deposition temperature of 225 °C was chosen because, based on reports using [Li(hmds)]_3_, the precursor [Li(^tBu^NHC)(hmds)] is expected to work well in this temperature regime.^[^ ^24^ ^]^ To ensure sufficient delivery of the precursor in the ALD chamber, a delivery temperature of 115 °C was applied. The typical ALD saturation behavior is seen for six or more consecutive 1‐s pulses (Figure 4a). A saturated growth per cycle (GPC) of 0.95 Å is reached, comparable to growth rates observed for Li(hmds) with ozone (∼0.7 Å) as well as with LiOtBu and tetraethylorthosilane using water as oxidant (∼0.8 Å) at the same temperature.^[^ ^11^, ^24^ ^]^ The slightly higher GPC that was observed herein hints at the more efficient packing of the surfaces adsorbates during the precursor pulse, the monomeric [Li(^tBu^NHC)(hmds)] exhibits when compared to other lithium sources. Additionally, GPCs notably exceeding 1 Å have been reported at higher temperatures or when oxygen plasma is used.^[^ ^11^, ^24^, ^59^ ^]^

Scanning electron microscopy (SEM) reveals island‐like surface features, which are uniformly distributed (Figure 4b)). Subsequent analysis by atomic force microscopy (AFM) reveals notable hill/valley like features with a root mean square roughness (*R*_q_) of 8.26 nm (Figure 4c). With this pronounced height difference, one must expect some of the Si substrate surface to be exposed. A similar surface topology has been observed for Li_2_Si_2_O_5_ prepared by hydrothermal synthesis, albeit the features were in the range of micrometers.^[^ ^60^ ^]^ An additional explanation for the morphology observed here can be attributed to the time taken between the depositions and the SEM and AFM measurements performed taking into account the sensitivity of lithium silicate to the ambient air as it can react with CO_2_ and absorb water.^[^ ^61^, ^62^ ^]^ The buildup of such a surface carbonate layer, paired with adsorption of water and the resulting structural changes, can lead to degradation of the layer leading to segregation of film areas into the features observed by SEM and AFM. Thin films deposited herein were found to be X‐ray amorphous, which is comparable to depositions performed with [Li(hmds)]_3_.^[^ ^24^ ^]^

Further, the composition and nature of the thin films were analyzed using complementary methods, namely Rutherford backscattering spectrometry (RBS), nuclear reaction analysis (NRA), X‐ray photoelectron spectrometry (XPS), and glow discharge optical emission spectrometry (GD‐OES). The standard practice of depositing on silicon wafers makes analysis complicated as the large penetration depth of RBS/NRA causes the substrate background to overlap the Si signal of the deposited film. To circumvent this effect, films deposited on glassy carbon (GC) were subjected to analysis, too. However, on GC, only RBS measurements are possible, preventing lithium from being determined on this substrate. Films produced with a total of 3 1‐s precursor pulses and 7 1‐s precursor pulses were investigated and showed a very close similarity in composition. From measurements performed on films grown on silicon, lithium and oxygen are present in an approximate 1:2 ratio. For films grown on GC, the presence of silicon can be confirmed with a Si to O ratio of approximately 1:2 for the 3 1‐s pulses and 1:4 for the 7 1‐s pulses. When looking at the total atom density determined by RBS/NRA on Si, for instance, the saturation trend of the precursor on the surface of the substrate is supported, producing 284 x 10^15^ atoms cm^−2^ for 3 1‐s precursor pulses compared to 394 x 10^15^ atoms cm^−2^ with 7 1‐s precursor pulses. If the silicon and lithium densities of the 7 1‐s pulses films from both substrates are correlated to their respective oxygen content, an approximate composition of 31 at.% Li, 13.5 at.% Si, 53 at.% O, and 2.5 at.% C can be estimated (Table 2). This is in good agreement with the metasilicate stoichiometry of Li_2_SiO_3_.

**Table 2:** Composition in atom densities (counts per cm^2^), determined by RBS/NRA of thin films grown by ALD with [Li(^tBu^NHC)(hmds)] and ozone on silicon and glassy carbon substrates. Greyed‐out values are arbitrary estimates only, which were implemented to reflect the expected composition.

Precursor Pulse Duration	Substrate	Li (10^15^ atoms cm^−2^)	Si (10^15^ atoms cm^−2^)	O (10^15^ atoms cm^−2^)	C (10^15^ atoms cm^−2^)	Approx. total atom densities (10^15^ atoms cm^−2^)
3 s	Si	86(8)	43	144(2)	11.1(4)	284
	GC	367	183(2)	381(4)		931
7 s	Si	120(9)	60	205(2)	9.7(4)	394
	GC	293	147(2)	577(5)		1017

Under the same deposition conditions, regarding the total atom densities, more material is deposited on the GC substrate than on Si. This effect may be explained by the choice of ozone as the second precursor, which can oxidize the glassy carbon surface, creating functional groups enabling stronger precursor adsorption within the first few ALD cycles. Further, roughening of the surface of GC and even further introducing mesoporosity can be results of the O_3_ treatment. This drastically increases the surface area and thus higher atom densities for oxygen can be detected.^[^ ^63^ ^]^

**Figure 5:**
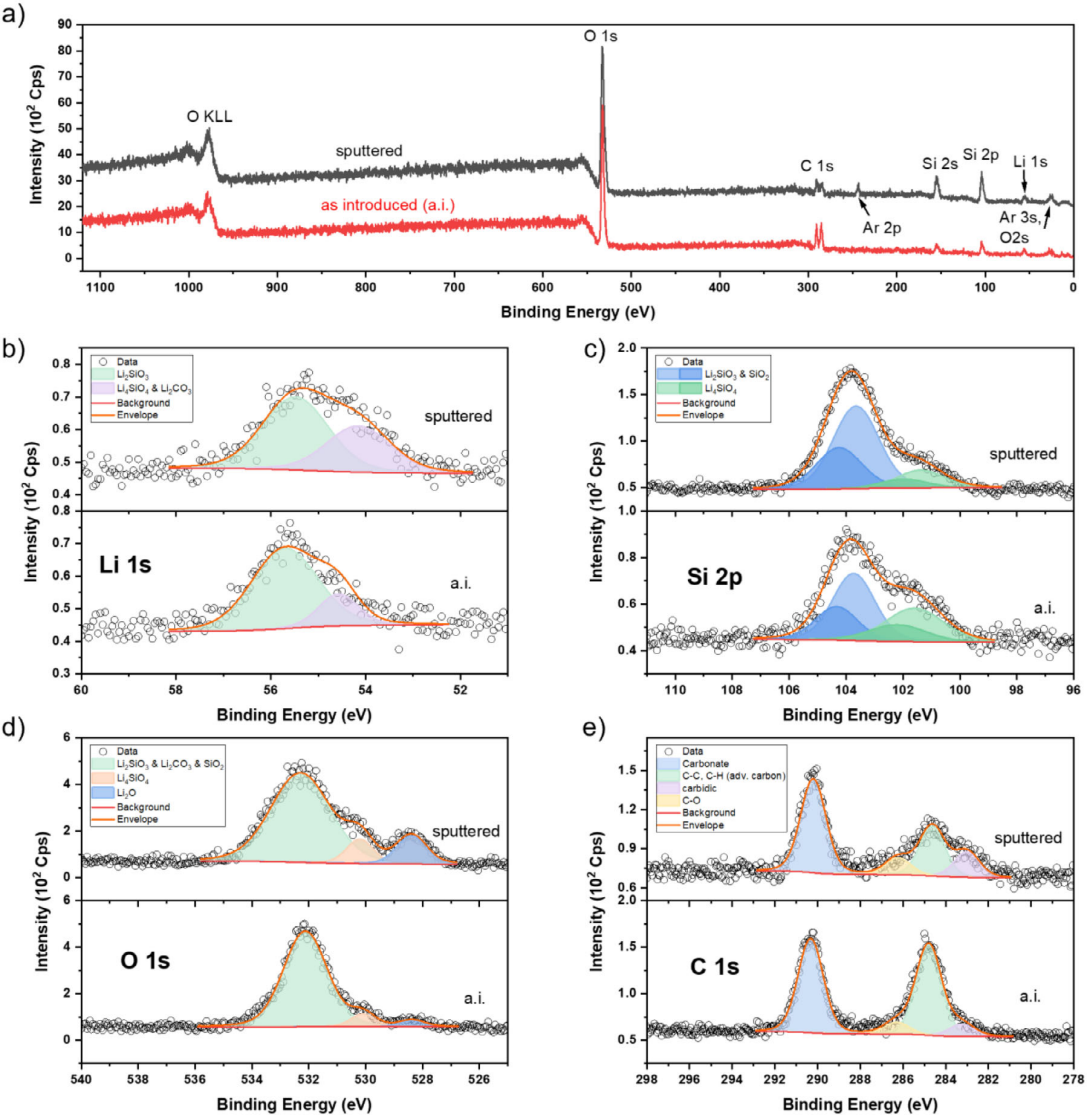
XPS spectra of an ALD film grown using [Li(^tBu^NHC)(hmds)] on silicon, before sputtering at the bottom of the respective graph marked with as introduced (a.i.) and after sputtering at the top marked with sputtered. a) Survey scans, b) Li 1s core level scans, c) Si 2p core level scans, d) O 1s core level scans, and e) C 1s core level scans. Please note the differences in the intensity scale after sputtering in (c) and (e).

To further determine which lithium silicate phase was deposited and to elucidate the chemical species on the film surfaces, XPS was conducted on specimens grown on silicon and GC. Samples were sputtered once to remove surface contaminations and the carbonate layer that is prone to form on lithium‐containing materials.^[^ ^64^, ^65^ ^]^ The relatively harsh sputtering conducted herein is expected to alter the sensitive thin films. Between films grown on silicon and GC, the same elements and chemical species were detected, see Figure 5 for Si and Figure SI 3 for GC. For lithium, two bonding modes were deconvoluted with a dominant peak at 55.6 eV, ascribed to the meta silicate Li_2_SiO_3_, and the less pronounced peak at 54.5 eV contributed by Li–O species attributable to ortho silicate phases, Li_4_SiO_4_, and lithium carbonate.^[^ ^66^, ^67^, ^68^ ^]^ After sputtering, the meta silicate peak is unchanged, but the lower binding energy peak shifts to a lower binding energy, 54.1 eV, and broadens slightly. Here, Li_2_O is assumed to additionally contribute to this peak. In the silicon 2p core level, two different peak sets were fitted. The one at 103.7 eV was ascribed to SiO_2_‐like species and the meta silicate Li_2_SiO_3_, while the second, less pronounced, lower binding energy peak at 101.4 eV corresponds to the orthosilicate Li_4_SiO_4_.^[^ ^66^, ^67^ ^]^ Between the Si and Li core levels, the dominant silicate phase can be identified as the metasilicate, Li_2_SiO_3_. The same two silicate phases are also seen in the O 1s core level fit, with three different peaks in total. The most prominent peak at 532.3 eV is attributed to overlapping contributions of Li_2_SiO_3_, SiO_2_, and carbonates.^[^ ^60^, ^69^ ^]^ At 530.1 eV, Li_4_SiO_4_ was observed, possibly together with hydroxide species.^[^ ^60^, ^69^ ^]^ While these two features remain similar in intensity and ratio, the third peak for Li_2_O at 528.4 eV becomes larger after sputtering.^[^ ^70^ ^]^ On the very surface of these thin films, the reactive Li_2_O does not persist, but reacts and forms carbonates. Its presence is only clearly revealed in the O 1s core level after sputtering, as in the Li 1s scan, more overlap occurs. The peak fit represents the contribution of Li_2_SiO_3_, SiO_2_, and carbonates, broadened post‐sputtering, indicative of a more diverse distribution of the species contained within it. The narrower appearance of the as‐introduced measurement signifies that it could be dominated by carbonate, while the silicates contribute more strongly within the body of the film. Analyzing the C 1s core level, the carbonate signal at 290.2 eV only reduces in intensity slightly after sputtering, but the aliphatic carbon signal (284.8 eV) reduces significantly. This is in line with the assumed carbonates seen with the O 1s spectra. Notably, a weak peak for carbidic carbon is seen at 283.0 eV, attributable to silicon carbide.^[^ ^71^ ^]^ This can be caused by remains of the hmds ligand. The NHC ligand has been shown to be the first leaving group by the DFT calculations and is expected to be evaporating facilely, but the hmds has been reported to dissociatively adsorb to the surface creating trimethylsilyl groups.^[^ ^27^ ^]^ If the combustion of those does not occur efficiently, for a fraction of them SiC can form. Additionally, the combustion of the methyl groups to CO_2_ introduces a possibility for the formation of lithium carbonates during the deposition process too, but GD‐OES analysis below shows carbonates to be present in the surface region mostly. Overall, XPS deduces a notable contribution of lithium carbonate in the surface region of these thin films.

When analyzing the survey scans, a notable observation is made: at first glance, all expected elements are seen for the as‐introduced spectra, which holds as well after sputtering with the addition of two argon signals, the 2p and 3s. Noble gas diffusion is a known phenomenon for silicate materials but is usually observed at elevated temperatures and pressures.^[^ ^72^ ^]^ Here, more likely, an implantation effect becomes visible.^[^ ^73^, ^74^ ^]^

**Figure 6:**
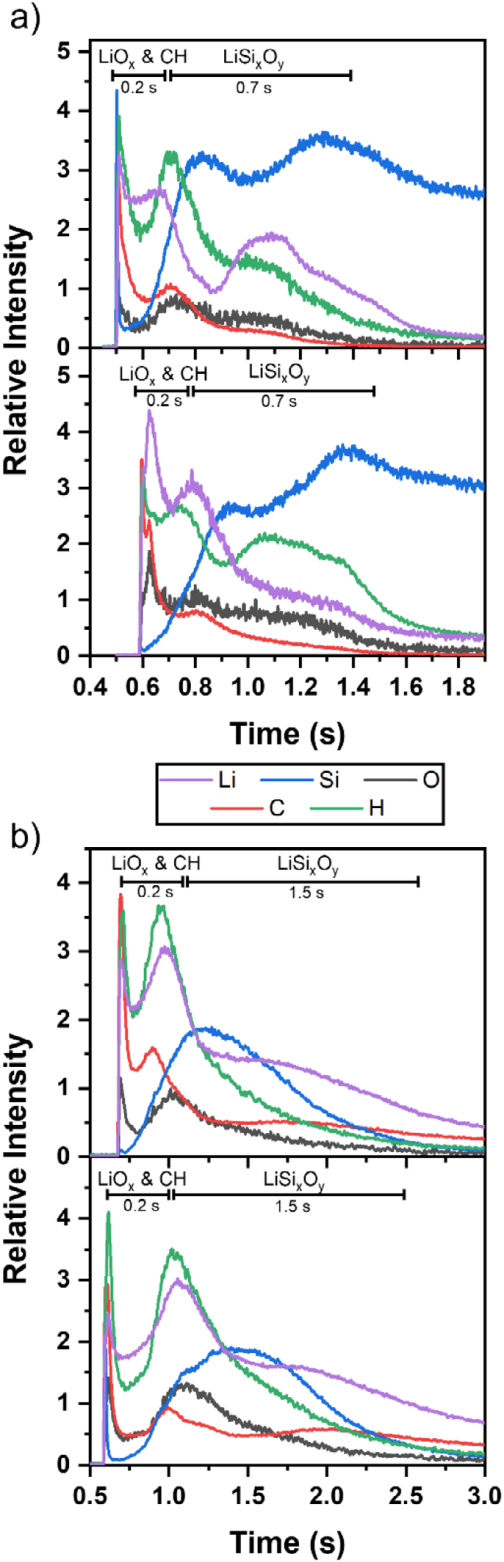
GD‐OES composition profiles of thin film samples grown by ALD with [Li(^tBu^NHC)(hmds)] on a) silicon and b) glassy carbon. Two scans for each substrate are shown to illustrate the reproducibility. Relative intensities for spectral lines of Li (671 nm), Si (288 nm), O (130 nm), C (165 nm), and H (121 nm) are shown. Additional elements are omitted for clarity. Full composition profiles are displayed in Figure SI 4.

To assess the composition, especially the content of Li, Si, and C simultaneously, as well as the extent of a surface carbonate layer further, GD‐OES was performed with thin films grown on silicon and GC (Figure 6). GD‐OES is a spectrometric technique that is used to investigate depth profiles as it is based on a plasma discharge process paired with spectral analysis of the characteristic optical emission lines in the plasma. It allows for simultaneous and fast detection of elements without profoundly altering the sample because the Ar^+^ sputtering ions possess less energy as compared to sputtering in XPS.^[^ ^75^ ^]^ The fast sputtering rates support the formation of non‐vertical sputtering crater walls, which can distort the depth profile resolution as well as variations in the sputtering rate of different elements.^[^ ^75^ ^]^ Additionally, the island‐like morphology of the thin films produced herein can affect the accuracy of the depth resolution. On silicon, a surface layer becomes evident by the elevated counts for Li, C, H, and O for an approximate 0.2 s discharge length in GD‐OES, likely to consist of lithium carbonate, lithium hydroxide, and LiO*_x_* species, which are expected to coexist in the surface near region. This is attributable to the formation of a carbonate and hydroxide layer upon exposure to ambient air, paired with adventitious carbon. While a degree of hydroxide formation was expected for lithium‐containing material, in this study, it is only evidenced by GD‐OES as it is the only technique employed that is able to directly detect hydrogen. With continued sputtering afterwards, a LiSi*_x_* O*_y_* region can be seen for about 0.7 s. Toward the Si substrate, the Li signal tails off gradually while no oxygen is detected anymore, which is indicative of Li diffusing into the surface region of the silicon.^[^ ^76^ ^]^ For the film grown on the GC substrate, a similar surface layer of carbonate, hydroxide, and LiO*_x_* species is observed with a comparable extent of 0.2 s of the sputtering time. Below, after this layer is sputtered away, a similar LiSi*_x_* O*_y_* layer as for the thin film grown on silicon is observed for about 1.5 s of the sputtering duration. Similar to the observation made for the sample deposited on Si, the lithium signal, after only very little oxygen is detected, attenuates, which can signify diffusion of lithium into the GC.^[^ ^77^ ^]^ The simultaneous lack of a high relative carbon signal is attributed to the low sputtering rate of the glassy carbon caused by its large ionization energy.^[^ ^78^ ^]^ The findings of GD‐EOS suggest that the sputtering step performed in the XPS experiments exposes the samples' surface somewhere at the interface of these two regions as notable amounts of carbonate were still detected alongside some Li_2_O contribution. Throughout all three compositional analysis methods, no nitrogen was detected within the films, indicative of the clean cleavage of the lithium–nitrogen and nitrogen–silicon bonds. Finally, all the compositional analyses reveal a mixed material with a surface layer of lithium carbonate and hydroxide, and the body of the films to be mainly metasilicate Li_2_SiO_3_. The surface carbonate region is identified as the main source of the carbon content that was detected by RBS/NRA. Some contributions of lithium oxide and the orthosilicate phase within the film must be acknowledged as well.

## Conclusions

For the first time, a very rare mononuclear Li compound [Li(^tBu^NHC)(hmds)] stabilized by carbene ligands has been isolated. The thermally stable [Li(^tBu^NHC)(hmds)] possesses highly promising thermal properties. Notably, it features a low melting point of 55 °C and high volatility, with a vapor pressure of 1 Torr at 130 °C. Especially, its lowered melting point allows for its use as a liquid precursor at the evaporation temperature in an ALD process.

DFT predicted the general structure of the precursor complex, which was confirmed by SC‐XRD. The structural analysis revealed greater flexibility in the coordination angle of the NHC ligand compared to similar ligands in coinage metals. Computational analysis of bond dissociation energies indicated that the NHC ligand is likely to cleave first during deposition. The analogous potassium complex shows a distinctly different dinuclear bridged solid state structure with the NHC ligands exhibiting a notable pitch.

The proof‐of‐concept ALD process investigated in this study demonstrated the applicability of [Li(^tBu^NHC)(hmds)] in ALD for lithium silicate. At a deposition temperature of 225 °C, saturated growth could be proven. RBS/NRA, XPS, and GD‐OES analysis revealed the dominant presence of the meta‐silicate phase, Li_2_SiO_3_. Appreciably low carbon concentrations were detected, which can mostly be ascribed to a carbonate surface layer formed upon contact with the ambient.

This study represents the first report of an ALD process using an NHC‐containing alkali metal precursor. By demonstrating the suitability and stabilizing effect of the NHC ligand for lithium precursors, this work paves the way for further exploration and development of NHC‐stabilized lithium complexes as precursors for ALD and other thin‐film deposition techniques in a range of relevant technological applications, including battery materials and catalysts.

## Supporting Information

Experimental detail ^1^H and ^13^C NMR spectra as well as crystallographic details for [Li(^tBu^NHC)(hmds)]. Synthetic and crystallographic details for [K(^tBu^NHC)(hmds)]_2_. XPS data for samples on glassy carbon and additional GD‐OES data (PDF). The authors have cited additional references within the Supporting Information.^[^ ^79^, ^80^, ^81^, ^82^, ^83^, ^84^, ^85^, ^86^, ^87^, ^88^, ^89^, ^90^, ^91^ ^]^

## Conflict of Interests

The authors declare no conflict of interest.

## Supporting information



Supporting information

## Data Availability

The data that support the findings of this study are available in the Supporting Information of this article.
