# Supplementary material for: Rare Mononuclear Lithium–Carbene Complex for Atomic Layer Deposition of Lithium Containing Thin Films
Source: Angew Chem Int Ed Engl. 2025 Sep 4;64(47):e202513066. doi: 10.1002/anie.202513066 (PMC12624310; doi:10.1002/anie.202513066)
Supplement: Supplementary file 1 — Supporting information [file ANIE-64-e202513066-s001.pdf]

Supporting Information

Rare Mononuclear Lithium Carbene Complex for Atomic Layer Deposition of Lithium-Containing Thin Films

Jorit Obenlüneschloß,[1] Nils Boysen,[2] Karl Rönnby,[3] Arbresha Muriqi,[3] Volker Hoffmann,[4, 5] Carlos Abad,[6] Detlef Rogalla,[7] Ulrike Brokmann,[8] Edda Rädlein,[8] Michael Nolan,[3] Anjana Devi*[1, 2, 4, 9]

[1]Inorganic Materials Chemistry, Ruhr University Bochum, Universitätsstr. 150, 44801 Bochum, Germany
[2]Fraunhofer IMS, Finkenstr. 61, 47057 Duisburg, Germany
[3]Tyndall National Institute, Lee Maltings, University College Cork, Cork T12 R5CP, Ireland
[4]Leibniz Institute for Solid State and Materials Research (IFW) Dresden e.V., Helmholtzstraße 20, Dresden 01069, Germany
[5]Spectruma Analytik GmbH, Fabrikzeile 21, Hof 95028, Germany
[6]Bundesanstalt für Materialforschung und -prüfung (BAM), 12205 Berlin
[7]RUBION, Ruhr University Bochum, Universitätsstr. 150, 44801 Bochum, Germany
[8]Institute of Materials Science and Engineering, Technical University Ilmenau, Ehrenbergstr. 29, 98693 Ilmenau, Germany
[9]Chair of Materials Chemistry, TU Dresden, Bergstr. 66, 01069 Dresden, Germany

Content

Experimental Section **2**
General Synthesis 2
Synthesis of 1,3-di-*tert*-butyl-imidazolin-2-ylidene lithium hexamethyldisilazide, [Li(^{tBu}NHC)(hmds)] 2
Synthesis of bis-1,3-di-*tert*-butyl-imidazolin-2-ylidene potassium hexamethyldisilazide, [K(^{tBu}NHC)(hmds)]$_2$ 2
Precursor Characterization 2
DFT Details 3
Thin Film Deposition and Characterization 3

NMR of [Li(^{tBu}NHC)(hmds)] **4**

NMR of [K(^{tBu}NHC)(hmds)]$_2$ **4**

Crystallographic details of [Li(^{tBu}NHC)(hmds)] and [K(^{tBu}NHC)(hmds)]$_2$ **5**
Additional crystallographic details for [Li(^{tBu}NHC)(hmds)] 6

XPS Spectra on Glassy Carbon **10**

GD-OES Analysis **11**

Experimental Section

General Synthesis

Syntheses were conducted in dried glassware under a dried argon atmosphere (AirLiquide, 99.995%) using conventional Schlenk techniques. Solvents were purified and dried using an MBraun-SPS-800 purification system and stored on molecular sieves (4 Å). Air- and moisture-sensitive compounds were handled in gloveboxes (MBraun). Deuterated benzene (benzene-d6) was purchased from Millipore, degassed before usage, and stored over 4 Å molecular sieves.
The synthesis of the starting reagents 1,3-di-*tert*-butyl imidazolium chloride and [Li(hmds)OEt_2], and [K(hmds)] was performed according to a literature known procedures.[79-81]

Synthesis of 1,3-di-*tert*-butyl-imidazolin-2-ylidene lithium hexamethyldisilazide, [Li(^{tBu}NHC)(hmds)]

Under an inert atmosphere, 1,3-di-*tert*-butyl imidazolium chloride (7.96 g, 36.2 mmol) and $[Li(hmds)OEt_2]_2$ (17.5 g, 36.2 mmol) were placed together in a Schlenk flask after which 100 ml of Et_2O were added. The reaction solution was heated to reflux for 12 hours. The solvent was removed, and the residual solid was extracted in warm hexane, filtered through a frit, reduced under vacuum and the product [Li(^{tBu}NHC)(hmds)] was purified by sublimation (60 °C, 10^{-2} mbar) and collected as a colorless solid (5.35 g, 15.3 mmol, 42%).
^{1}H NMR (200 MHz, C_6D_6): δ 2.69 [s, 4H, $N(CH_2)_2N$], δ 1.06 [s, 18H, $NC(CH_3)_3$], δ 0.43 [s, 18H, $N(Si(CH_3)_3)_2$].
^{13}C NMR (75 MHz, C_6D_6): δ 213.7 [NCN], δ 54.2 [$NC(CH_3)_3$], δ 44.5 [$N(CH_2)_2N$], δ 29.6 [$NC(CH_3)_3$], δ 6.3 [$N(Si(CH_3)_3)_2$].
EA calculated (%): N 12.02, C 58.40, H 11.53; found: N 12.34, C 56.20, H 11.06.

Synthesis of bis-1,3-di-*tert*-butyl-imidazolin-2-ylidene potassium hexamethyldisilazide, $[K(^{tBu}NHC)(hmds)]_2$

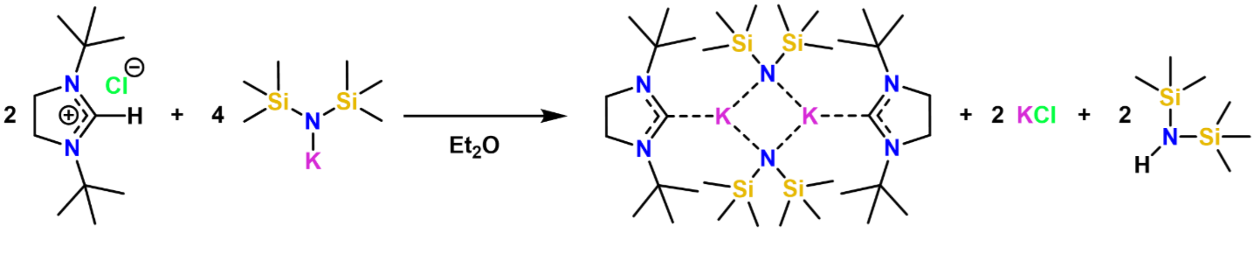

$[K(^{tBu}NHC)(hmds)]_2$

Scheme SI 1: Synthesis of $[K(^{tBu}NHC)(hmds)]_2$.

$[K(^{tBu}NHC)(hmds)]_2$ was prepared following a similar procedure to for [Li(^{tBu}NHC)(hmds)]. Under an inert atmosphere, 1,3-di-*tert*-butyl imidazolium chloride (1.0 g, 4.55 mmol) and [K(hmds)] (1.9 g, 9.56 mmol, 2 eq.) were placed in a Schlenk flask together, after which 50 mL of Et_2O was added. The reaction solution was heated to reflux for 20 hours. The solvent was removed, and the residual solid was extracted in warm hexane, filtered through a frit, and reduced under vacuum. The product $[K(^{tBu}NHC)(hmds)]_2$ was crystallized from a saturated hexane solution (-35 °C) and collected as a colorless solid (0.77 g, 2.01 mmol, 44 %).
^{1}H NMR (200 MHz, C_6D_6): δ 3.04 [s, 4H, $N(CH_2)_2N$], δ 1.37 [s, 18H, $NC(CH_3)_3$], δ 0.14 [s, 18H, $N(Si(CH_3)_3)_2$].

Precursor Characterization

^{1}H and ^{13}C NMR spectra were recorded on a Bruker DPX 200 instrument and referenced to the internal tetramethyl silane (TMS) standard (TMS, δ = 0.00 ppm). Elemental analysis (EA) was conducted with a vario micro cube tool (Elementar Analysesysteme) in the CHNS analysis mode. TG and stepped iso-TG measurements were conducted on a Netzsch STA-409 device in a temperature range of 30 to 550 °C. The TG experiments were run under an argon atmosphere with a sample size of ≈10 mg for the dynamic measurement, a heating rate of 5 K min^{-1}, and a nitrogen flow rate (AirLiquide, 99.999%) of 300 mL min^{-1}. For the stepped isothermal experiment, a sample of ≈25 mg was submitted, and the temperature increased in 10 °C steps, whereby the heating rate was set to 40 °C min^{-1} and held constant for 10 min at each step.

The evaporation rates were determined by a linear fit of the mass loss under consideration of the surface area of the crucible. A single crystal specimen of [Li(^{tBu}NHC)(hmds)] and of [K(^{tBu}NHC)(hmds)]$_2$ was crystallized from concentrated hexane solution at −35 °C. A suitable crystal was selected under a microscope in perfluoro-ether oil and mounted to a Synergy S diffractometer (Oxford Rigaku) with a HyPix 600 HE detector with a flexible loop. The crystals were cooled to 100 K during data collection. Structure solution was undertaken using the Olex2 software suite,[82] with the SHELXT structure solution program using Intrinsic Phasing and refined with the SHELXL refinement package using Least Squares minimization.[83,84] Deposition Numbers 2454532 (for [Li(^{tBu}NHC)(hmds)]) and 2461832 (for [K(^{tBu}NHC)(hmds)]$_2$) contain the supplementary crystallographic data for this paper. These data are provided free of charge by the joint Cambridge Crystallographic Data Centre and Fachinformationszentrum Karlsruhe via http://www.ccdc.cam.ac.uk/structures.

DFT Details

DFT calculations were performed using the TURBOMOLE software suite.[85,86] The hybrid functional PBE0 was used to approximate the exchange-correlation potential.[87] The basis set used was a triple zeta basis set, denoted def2-TZVP.[88,89] Initial studies using a smaller split valence basis set (def2-SVP) yielded bond lengths that deviated significantly from the experimental values. The convergence criteria for the electronic SCF cycles were 10^{-6} Ha. The molecular structures were optimized using redundant internal coordinates and a convergence criterion of the energy gradient less than 10^{-3} Ha/Bohr. The bond dissociation energy was calculated by the energy difference between the fragments after bond dissociation and the full molecule, Equation 1.

$$\Delta_{BDE}E_{NHC/hmds} = E_{Li(hmds)/Li(NHC)} + E_{NHC/hmds} - E_{Li(NHC)(hmds)} \tag{1}$$

Thin Film Deposition and Characterization

ALD experiments were performed in a custom-built thermal ALD reactor (modularflow) at 225 °C. The vapor draw precursor bubbler was held at 115 °C for [Li(^{tBu}NHC)(hmds)]. Ozone was pulsed for 5 seconds, provided by an ozone generator (Anseros, COM-AD-01) supplied with 50 sccm oxygen at 0.5 bar (AirLiquide, 99.995 %). Precursor and ozone pulses were separated by 10-second nitrogen purges each. Depositions were carried out with an N_2 flow of 10 sccm at a base pressure of 1.7 mbar on 1-inch prime CZ-Si(100) p-type wafers with native oxide (MicroChemicals) and glassy carbon plates (SIGRADUR® G, HTW Germany). Film thicknesses were determined by spectroscopic ellipsometry (J.A. Woollam VASE ellipsometer) using a Xenon light source with an angle of incidence of 70° and a polarization of 30°.

AFM measurements were performed in peak force tapping mode on a DimensionEdge device (Bruker).

SEM data were recorded using a JEOL JSM-7200F microscope with a field emission cathode, an accelerating voltage of 20 kV, and a beam current of 9 nA.

RBS/NRA measurements were done at the Central Unit for Ion Beams and Radionuclides (RUBION) at the Ruhr University Bochum, with a $^4He^+$ ion beam of 2.0 MeV. NRA was executed using deuteron ion beams with 1.0 MeV. For processing and analysis of RBS and NRA raw data, the SIMNRA program was utilized.[90]

XPS was performed with a VersaProbe 5000 spectrometer from PHI (Physical Electronics) at the chair of Experimentalphysik II (Ruhr University Bochum, Germany). An Al anode material with Kα radiation of 1486.7 eV and a spot size of 200 μm in diameter with a pass energy of 23.5 eV was used. Survey and core-level scans were recorded for as-introduced and sputtered films on silicon and glassy carbon substrates. Sputtering was conducted with Ar^+ ions accelerated at 2 kV with a 2x2 mm spot size and sputtering times of 60 seconds. The binding energy scale charge correction was done with the non-sputtered surfaces to the signal of adventitious carbon at 284.8 eV. The overall very similar peak shapes, fitted components, and peak positions for films deposited on both investigated substrates (Si, GC) justify the use of this method. Deconvolution was done using CasaXPS, fitting a Shirley background and appropriate components.[91]

GD-OES depth profiles were measured in the joint GD-OES lab of BAM Berlin and IFW Dresden, using a GDA750 HR (Spectruma Analytik GmbH, Hof, Germany). The samples were continuously sputtered in radio-frequency mode at 6.78 MHz, 500 V anode voltage, and 2.7 hPa Ar pressure. A modified universal sample unit of Spectruma was applied, where the samples do not act as a seal to the atmosphere. This sputtering source is of Grimm type with a 2.5 mm diameter anode and uses water cooling from the backside.

NMR of [Li(^{tBu}NHC)(hmds)]

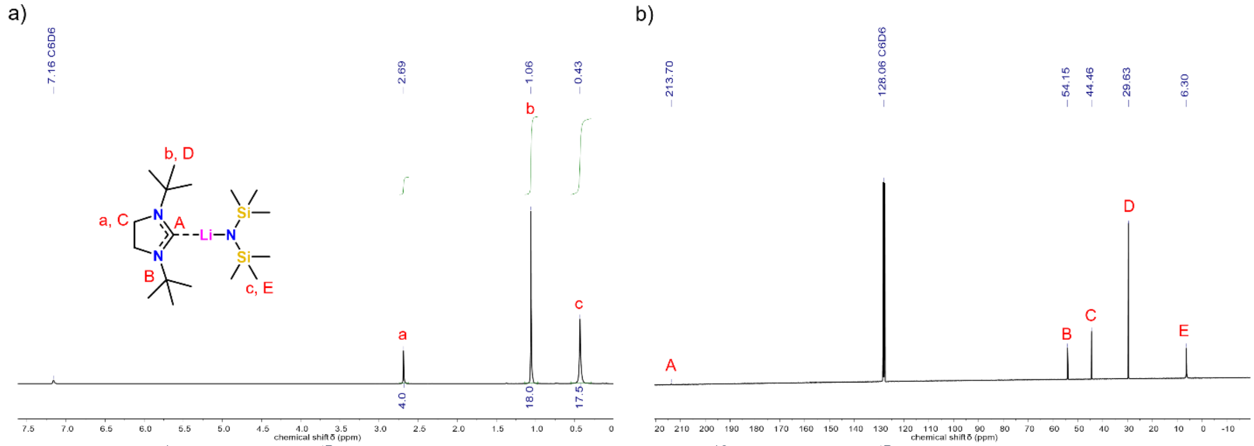

Figure SI 1: a) ^{1}H NMR of [Li(^{tBu}NHC)(hmds)] with assignment. b) ^{13}C NMR of [Li(^{tBu}NHC)(hmds)] with assignment in capital letters in a).

NMR of [K(^{tBu}NHC)(hmds)]$_2$

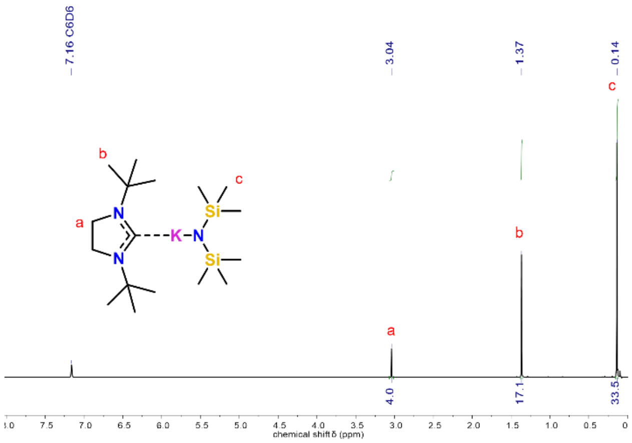

Figure SI 2: ^{1}H NMR of [Li(^{tBu}NHC)(hmds)] with assignment. The peak for c is overlapping with the peak for free K(hmds) that is still present in the sample.

Crystallographic details of [Li(^{tBu}NHC)(hmds)] and [K(^{tBu}NHC)(hmds)]$_2$

Table SI 1: Crystal data and structure refinement for [Li(^{tBu}NHC)(hmds)] and [K(^{tBu}NHC)(hmds)]$_2$.

Identification code	[Li(^{tBu}NHC)(hmds)]	[K(^{tBu}NHC)(hmds)]$_2$
Empirical formula	$C_{17}H_{40}LiN_3Si_2$	$C_{34}H_{80}K_2N_6Si_4$
Formula weight	349.64	763.60
Temperature/K	100.01(10)	149.99(10)
Crystal system	monoclinic	triclinic
Space group	$P2_1/n$	P-1
a/Å	11.90850(10)	10.6915(4)
b/Å	11.9169(2)	11.5333(5)
c/Å	16.6175(2)	11.8693(6)
α/°	90	69.802(4)
β/°	101.3670(10)	67.055(4)
γ/°	90	65.536(4)
Volume/Å^3	2311.97(5)	1196.92(11)
Z	4	2
ρ_{calc}g/cm^3	1.004	1.059
μ/mm^{-1}	1.387	2.910
F(000)	776.0	420.0
Crystal size/mm^3	0.207 × 0.133 × 0.102	0.130 × 0.216 × 0.283
Radiation	Cu Kα (λ = 1.54184)	Cu Kα (λ = 1.54184)
2Θ range for data collection/°	8.404 to 155.37	8.292 to 153.354
Index ranges	-15 ≤ h ≤ 13, -15 ≤ k ≤ 11, -20 ≤ l ≤ 21	-11 ≤ h ≤ 13, -14 ≤ k ≤ 14, -14 ≤ l ≤ 14
Reflections collected	29079	19140
Independent reflections	4841 [R_{int} = 0.0464, R_{sigma} = 0.0290]	4740 [R_{int} = 0.0545, R_{sigma} = 0.0371]
Data/restraints/parameters	4841/0/242	4740/0/261
Goodness-of-fit on F^2	1.103	1.088
Final R indexes [I>=2σ (I)]	R_1 = 0.0378, wR_2 = 0.1008	R_1 = 0.0472, wR_2 = 0.1278
Final R indexes [all data]	R_1 = 0.0416, wR_2 = 0.1033	R_1 = 0.0564, wR_2 = 0.1353
Largest diff. peak/hole / e Å^{-3}	0.29/-0.31	0.41/-0.43

Additional crystallographic details for [Li(^{tBu}NHC)(hmds)]

Table SI 2: Fractional Atomic Coordinates (×10^4) and Equivalent Isotropic Displacement Parameters (Å^2×10^3) for [Li(^{tBu}NHC)(hmds)]. U_{eq} is defined as 1/3 of the trace of the orthogonalized U_{IJ} tensor.

Atom	*x*	*y*	*z*	U(eq)
Si1	4635.8(3)	4324.4(3)	1486.1(2)	20.52(10)
Si2	5219.2(3)	2658.7(3)	2868.7(2)	23.89(11)
N3	968.6(9)	3964.0(9)	3469.6(7)	20.9(2)
N2	244.4(9)	3238.8(10)	2288.3(7)	23.1(2)
N1	4334.1(9)	3535.6(9)	2256.6(7)	22.4(2)
C1	1206.8(10)	3657.2(11)	2745.9(8)	20.8(3)
C5	1772.1(10)	4471.1(11)	4166.1(8)	21.3(3)
C2	-206.6(11)	3653.8(12)	3556.1(9)	26.5(3)
C11	1340.4(12)	5634.2(12)	4340.6(9)	28.1(3)
C4	93.8(12)	2838.1(13)	1430.4(8)	27.8(3)
C9	2956.7(12)	4592.3(14)	3960.6(9)	30.1(3)
C17	3611.8(13)	5543.5(12)	1264.3(10)	30.8(3)
C8	-290.0(13)	1611.1(13)	1394.5(9)	31.7(3)
C3	-755.8(11)	3338.1(14)	2684.2(9)	31.0(3)
C16	4498.4(15)	3527.0(13)	495.0(9)	34.5(3)
C10	1848.2(12)	3724.7(13)	4922.6(9)	31.1(3)
C14	4369.9(16)	1610.0(13)	3358.3(11)	39.8(4)
C12	6185.2(13)	1789.9(14)	2341.2(10)	35.6(3)
C15	6123.7(12)	4943.3(14)	1699.4(10)	34.4(3)
C7	-823.5(16)	3550.2(14)	882.0(10)	39.9(4)
C13	6207.6(15)	3387.1(14)	3740.6(10)	39.4(4)
C6	1220.7(16)	2923(2)	1132.9(11)	47.0(5)
Li1	2834(2)	3698(2)	2427.8(16)	33.0(5)

Table SI 3: Anisotropic Displacement Parameters (Å^2×10^3) for [Li(^{tBu}NHC)(hmds)]. The Anisotropic displacement factor exponent takes the form: $-2\pi^2[h^2a^{*2}U_{11}+2hka^*b^*U_{12}+\ldots]$.

Atom	U_{11}	U_{22}	U_{33}	U_{23}	U_{13}	U_{12}
Si1	20.10(18)	20.92(18)	22.62(19)	-0.26(12)	9.30(13)	-0.80(12)
Si2	25.07(19)	22.78(19)	23.40(19)	-0.57(13)	3.72(14)	0.93(13)
N3	15.9(5)	25.2(5)	23.5(5)	-3.6(4)	8.1(4)	-2.4(4)
N2	18.0(5)	28.2(6)	24.0(5)	-5.1(4)	6.5(4)	-3.1(4)
N1	19.4(5)	26.3(5)	23.5(5)	0.4(4)	9.4(4)	0.6(4)
C1	20.4(6)	20.8(6)	22.0(6)	-1.9(5)	6.1(5)	-0.8(5)
C5	19.1(6)	24.6(6)	20.8(6)	-2.6(5)	5.7(5)	-0.4(5)
C2	18.5(6)	33.1(7)	30.5(7)	-4.5(6)	11.3(5)	-4.3(5)
C11	28.7(7)	26.3(7)	28.6(7)	-6.3(5)	3.6(5)	1.7(5)
C4	26.5(7)	34.1(7)	23.2(7)	-7.0(5)	5.9(5)	-5.6(6)
C9	20.0(6)	40.5(8)	30.7(7)	-12.0(6)	7.3(5)	-7.2(6)
C17	29.9(7)	26.1(7)	37.2(8)	3.9(6)	8.5(6)	3.3(6)
C8	30.6(7)	31.3(7)	32.0(7)	-9.0(6)	3.3(6)	1.4(6)
C3	19.1(6)	41.6(8)	34.0(8)	-12.4(6)	8.8(5)	-4.7(6)
C16	53.5(9)	28.3(7)	26.2(7)	-0.8(6)	18.9(7)	-5.6(7)
C10	28.6(7)	34.8(8)	28.7(7)	5.8(6)	2.5(5)	1.5(6)
C14	53.7(10)	28.2(7)	40.3(9)	7.2(6)	15.8(7)	-0.6(7)

Atom	U_{11}	U_{22}	U_{33}	U_{23}	U_{13}	U_{12}
C12	26.7(7)	34.8(8)	43.5(9)	-6.2(7)	2.6(6)	7.3(6)
C15	26.4(7)	36.6(8)	42.5(8)	3.7(6)	12.4(6)	-6.0(6)
C7	52.5(10)	34.4(8)	29.0(8)	0.0(6)	-1.7(7)	-6.5(7)
C13	45.3(9)	35.2(8)	31.6(8)	-3.0(6)	-7.0(7)	1.9(7)
C6	39.8(9)	74.3(13)	31.6(8)	-24.0(9)	18.0(7)	-22.8(9)
Li1	22.6(11)	46.1(15)	33.7(13)	-4.6(11)	14.2(10)	-3.0(10)

Table SI 4: Bond Lengths for [Li(^{tBu}NHC)(hmds)].

Atom	Atom	Length/Å	Atom	Atom	Length/Å
Si1	N1	1.6832(11)	N2	C4	1.4805(17)
Si1	C17	1.8858(15)	N2	C3	1.4746(17)
Si1	C16	1.8805(15)	N1	Li1	1.874(2)
Si1	C15	1.8872(15)	C1	Li1	2.107(3)
Si1	Li1	2.989(2)	C5	C11	1.5259(18)
Si2	N1	1.6778(11)	C5	C9	1.5223(18)
Si2	C14	1.8897(16)	C5	C10	1.5280(19)
Si2	C12	1.8871(16)	C2	C3	1.515(2)
Si2	C13	1.8884(16)	C4	C8	1.530(2)
Si2	Li1	3.053(3)	C4	C7	1.534(2)
N3	C1	1.3398(16)	C4	C6	1.522(2)
N3	C5	1.4776(16)	C9	Li1	2.739(3)
N3	C2	1.4816(16)	C6	Li1	2.746(3)
N2	C1	1.3405(16)			

Table SI 5: Bond Angles for [Li(^{tBu}NHC)(hmds)].

Atom	Atom	Atom	Angle/˚	Atom	Atom	Atom	Angle/˚
N1	Si1	C17	110.62(6)	N3	C5	C11	109.27(10)
N1	Si1	C16	112.91(6)	N3	C5	C9	110.41(10)
N1	Si1	C15	114.12(6)	N3	C5	C10	109.30(11)
N1	Si1	Li1	34.85(6)	C11	C5	C10	109.87(11)
C17	Si1	C15	106.41(7)	C9	C5	C11	108.56(11)
C17	Si1	Li1	77.97(7)	C9	C5	C10	109.42(12)
C16	Si1	C17	106.07(7)	N3	C2	C3	101.96(10)
C16	Si1	C15	106.16(7)	N2	C4	C8	108.93(12)
C16	Si1	Li1	112.62(7)	N2	C4	C7	109.31(12)
C15	Si1	Li1	138.05(7)	N2	C4	C6	110.13(11)
N1	Si2	C14	110.32(7)	C8	C4	C7	109.48(12)
N1	Si2	C12	115.33(7)	C6	C4	C8	109.06(14)
N1	Si2	C13	113.64(7)	C6	C4	C7	109.91(15)
N1	Si2	Li1	32.67(6)	C5	C9	Li1	106.84(10)
C14	Si2	Li1	79.62(7)	N2	C3	C2	102.25(10)
C12	Si2	C14	105.32(8)	C4	C6	Li1	107.05(11)
C12	Si2	C13	105.30(7)	Si1	Li1	Si2	59.79(4)
C12	Si2	Li1	137.28(7)	N1	Li1	Si1	30.89(6)
C13	Si2	C14	106.21(8)	N1	Li1	Si2	28.90(6)
C13	Si2	Li1	114.05(7)	N1	Li1	C1	170.85(17)

Atom	Atom	Atom	Angle/˚	Atom	Atom	Atom	Angle/˚
C1	N3	C5	126.56(10)	N1	Li1	C9	107.67(12)
C1	N3	C2	112.97(10)	N1	Li1	C6	112.78(12)
C5	N3	C2	120.28(10)	C1	Li1	Si1	158.06(13)
C1	N2	C4	126.44(11)	C1	Li1	Si2	142.07(13)
C1	N2	C3	113.11(11)	C1	Li1	C9	70.04(8)
C3	N2	C4	120.02(11)	C1	Li1	C6	69.70(8)
Si1	N1	Li1	114.26(10)	C9	Li1	Si1	118.51(9)
Si2	N1	Si1	127.31(6)	C9	Li1	Si2	93.33(8)
Si2	N1	Li1	118.43(10)	C9	Li1	C6	139.50(10)
N3	C1	N2	107.58(10)	C6	Li1	Si1	97.89(8)
N3	C1	Li1	125.94(11)	C6	Li1	Si2	121.73(10)
N2	C1	Li1	126.29(12)				

Table SI 6: Torsion Angles for [Li(^{tBu}NHC)(hmds)].

A	B	C	D	Angle/˚	A	B	C	D	Angle/˚
Si1	N1	Li1	Si2	-179.51(14)	C4	N2	C1	N3	177.37(12)
Si1	N1	Li1	C9	-116.31(10)	C4	N2	C1	Li1	-7.5(2)
Si1	N1	Li1	C6	65.69(15)	C4	N2	C3	C2	174.64(12)
Si2	N1	Li1	Si1	179.51(14)	C17	Si1	N1	Si2	-157.89(8)
Si2	N1	Li1	C9	63.19(14)	C17	Si1	N1	Li1	21.57(12)
Si2	N1	Li1	C6	-114.81(12)	C8	C4	C6	Li1	-117.24(13)
N3	C5	C9	Li1	-2.19(14)	C3	N2	C1	N3	4.92(16)
N3	C2	C3	N2	13.73(14)	C3	N2	C1	Li1	-179.92(14)
N2	C4	C6	Li1	2.25(18)	C3	N2	C4	C8	-66.27(16)
C1	N3	C5	C11	118.42(14)	C3	N2	C4	C7	53.32(17)
C1	N3	C5	C9	-0.91(18)	C3	N2	C4	C6	174.16(15)
C1	N3	C5	C10	-121.32(14)	C16	Si1	N1	Si2	83.42(10)
C1	N3	C2	C3	-12.55(15)	C16	Si1	N1	Li1	-97.12(12)
C1	N2	C4	C8	121.76(14)	C10	C5	C9	Li1	118.15(12)
C1	N2	C4	C7	-118.66(15)	C14	Si2	N1	Si1	-159.56(9)
C1	N2	C4	C6	2.2(2)	C14	Si2	N1	Li1	21.01(13)
C1	N2	C3	C2	-12.38(16)	C12	Si2	N1	Si1	-40.42(11)
C5	N3	C1	N2	-179.81(12)	C12	Si2	N1	Li1	140.14(12)
C5	N3	C1	Li1	5.0(2)	C15	Si1	N1	Si2	-37.93(11)
C5	N3	C2	C3	172.19(11)	C15	Si1	N1	Li1	141.53(11)
C2	N3	C1	N2	5.29(15)	C7	C4	C6	Li1	122.73(13)
C2	N3	C1	Li1	-169.89(13)	C13	Si2	N1	Si1	81.29(11)
C2	N3	C5	C11	-67.02(15)	C13	Si2	N1	Li1	-98.14(13)
C2	N3	C5	C9	173.65(12)	Li1	Si1	N1	Si2	-179.46(16)
C2	N3	C5	C10	53.24(15)	Li1	Si2	N1	Si1	179.44(17)
C11	C5	C9	Li1	-121.94(11)					

Table SI 7: Hydrogen Atom Coordinates (Å×10^4) and Isotropic Displacement Parameters (Å^2×10^3) for [Li(^{tBu}NHC)(hmds)].

**Atom**	***x***	***y***	***z***	**U(eq)**
H2A	-597.94	4282.11	3750.16	32
H2B	-199.48	3024.74	3927.49	32
H11A	1309.3	6103.42	3867.16	42
H11B	1851.88	5959.31	4800.53	42
H11C	588.62	5571.61	4464.06	42
H17A	2840.25	5267.09	1131.03	46
H17B	3776.69	5966.12	809.6	46
H17C	3697.27	6017.98	1739.38	46
H8A	268.76	1170.6	1756.02	48
H8B	-364.4	1336.86	843.26	48
H8C	-1015.07	1555.63	1561.54	48
H3A	-1165.98	2632.53	2666.93	37
H3B	-1277.4	3917.42	2426.57	37
H16A	4999.45	2888.43	575.98	52
H16B	4703.83	4006.75	82.93	52
H16C	3721.64	3278.71	319.99	52
H10A	1103.25	3656.03	5056.99	47
H10B	2367.26	4053.92	5376.66	47
H10C	2121.79	2995.11	4809.36	47
H14A	3805.55	1998.27	3593.21	60
H14B	4876.86	1206.81	3781	60
H14C	3995.93	1092.21	2949.35	60
H12A	5728.17	1305.59	1944.94	53
H12B	6685.46	1345.03	2741.09	53
H12C	6633.41	2277.19	2069.07	53
H15A	6240.82	5355.34	2205.47	52
H15B	6208.66	5438.64	1259.7	52
H15C	6678.38	4350.83	1741.39	52
H7A	-1540.94	3463.28	1055.94	60
H7B	-904.78	3308.2	322.48	60
H7C	-598.97	4325.07	924.28	60
H13A	6680.3	3912.93	3522.35	59
H13B	6683.47	2840.55	4069.2	59
H13C	5761.37	3778.22	4073.61	59
H9A	3299(15)	3847(16)	3885(11)	32(4)
H9B	3458(15)	4949(15)	4425(12)	34(4)
H6A	1430(17)	3704(18)	1114(12)	43(5)
H9C	2938(15)	5092(16)	3482(12)	37(5)
H6B	1835(19)	2440(18)	1478(14)	53(6)
H6C	1100(20)	2660(20)	553(17)	71(7)

XPS Spectra on Glassy Carbon

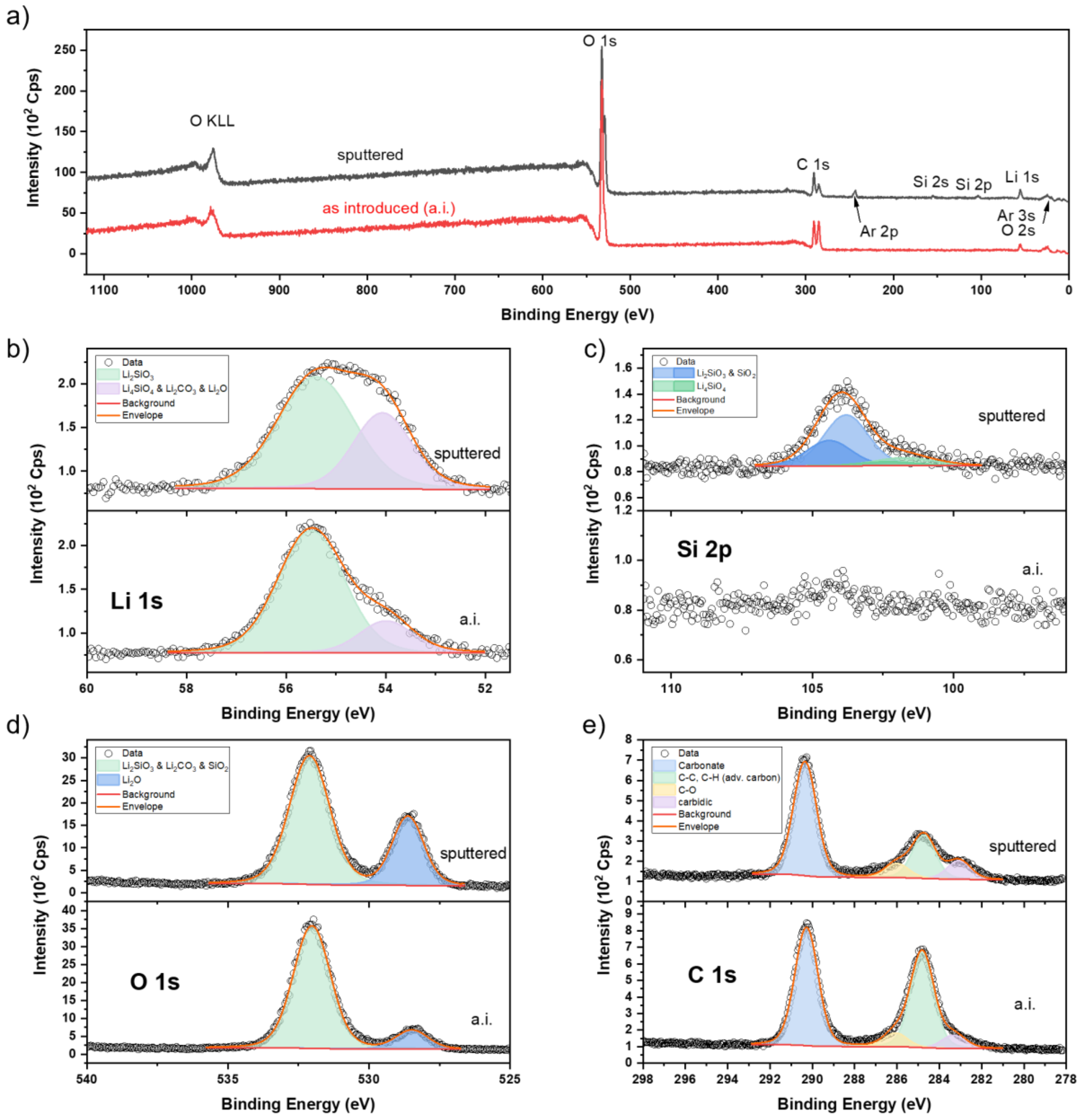

Figure SI 3: XPS spectra of an ALD film grown using [Li(^{tBu}NHC)(hmds)] at 225 °C on glassy carbon. Before sputtering at the bottom of the respective graph marked as introduced (a.i.) and after sputtering at the top marked with sputtered. a) Survey scans, b) Li 1s core level scans, c) Si 2p core level scans, d) O1s core level scans, and e) C 1s core level scans. Please note the differences in the intensity scale after sputtering in c) and e).

GD-OES Analysis

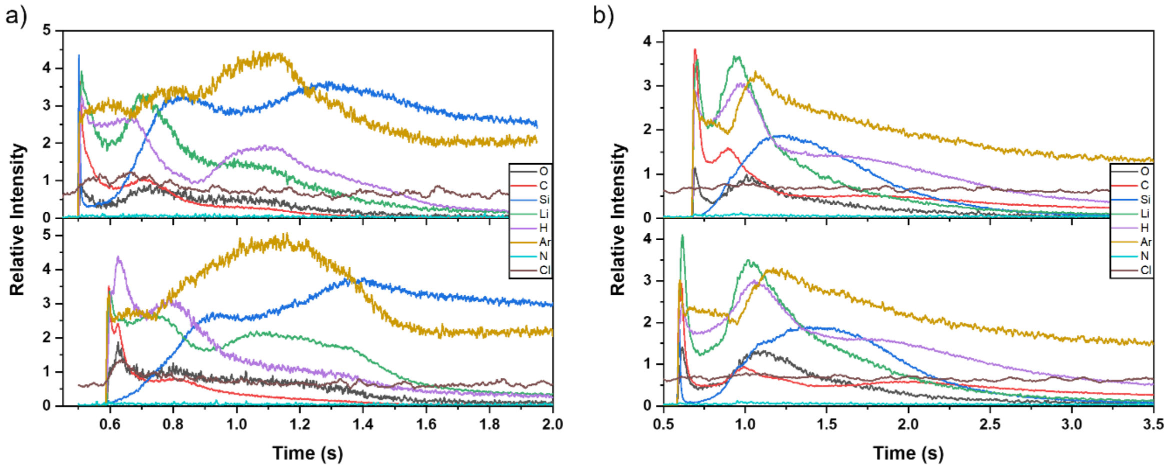

Figure SI 4: Full glow discharge optical emission spectrometry (GD-OES) composition profiles of thin film samples grown by ALD at 225 °C with [Li(^{tBu}NHC)(hmds)] on a) silicon, and b) glassy carbon. Relative intensities for spectral lines of O (130 nm), C (165 nm), Si (288 nm), Li (671 nm), H (121 nm), Ar (415 nm), N (149 nm), and Cl (134 nm) are shown.

For the thin film sample grown on silicon Figure SI 4 a), bottom, the measurement shows a signal for chlorine in the surface region. This outlier can be attributed to the possible handling of hydrochloric acid in the vicinity of the sample and is not a result of the deposition process.
